# Anomaly Detection in Industrial IoT Using Distributional Reinforcement Learning and Generative Adversarial Networks

**DOI:** 10.3390/s22218085

**Published:** 2022-10-22

**Authors:** Hafsa Benaddi, Mohammed Jouhari, Khalil Ibrahimi, Jalel Ben Othman, El Mehdi Amhoud

**Affiliations:** 1 Laboratory of Research in Informatics (LaRI), Faculty of Sciences, Ibn Tofail University, Kenitra 14000, Morocco; 2 School of Computer Science, Mohammed VI Polytechnic University, Ben Guerir 43150, Morocco; 3 L2S Laboratory, Paris-Saclay University, CNRS, Centralesupelec, 91190 Gif-sur-Yvette, France

**Keywords:** Industrial Internet of Things, anomaly detection, DS2OS, Generative Adversarial Network, Distributional Reinforcement Learning, adversarial machine learning

## Abstract

Anomaly detection is one of the biggest issues of security in the Industrial Internet of Things (IIoT) due to the increase in cyber attack dangers for distributed devices and critical infrastructure networks. To face these challenges, the Intrusion Detection System (IDS) is suggested as a robust mechanism to protect and monitor malicious activities in IIoT networks. In this work, we suggest a new mechanism to improve the efficiency and robustness of the IDS system using Distributional Reinforcement Learning (DRL) and the Generative Adversarial Network (GAN). We aim to develop realistic and equilibrated distribution for a given feature set using artificial data in order to overcome the issue of data imbalance. We show how the GAN can efficiently assist the distributional RL-based-IDS in enhancing the detection of minority attacks. To assess the taxonomy of our approach, we verified the effectiveness of our algorithm by using the Distributed Smart Space Orchestration System (DS2OS) dataset. The performance of the normal DRL and DRL-GAN models in binary and multiclass classifications was evaluated based on anomaly detection datasets. The proposed models outperformed the normal DRL in the standard metrics of accuracy, precision, recall, and F1 score. We demonstrated that the GAN introduced in the training process of DRL with the aim of improving the detection of a specific class of data achieves the best results.

## 1. Introduction

The Internet of Things (IoT) refers to a network of sensors and computing devices with a shared purpose of solving issues and delivering new services. The IoT is a rapidly progressing field of technological development for connecting devices or things. The IoT operates through the deployment of hundreds of smart environments in living and industrial settings established to deal with livelihood, fabrication, power consumption, and business requirements [1]. An important element of the IoT environment is the sensor, which collects data and then sends them to the central agency for further processing. The intelligent devices interact with each other via the internet to interchange data. Through the use of sensors, smart environments aim to achieve an improvement in the quality of life of human beings while increasing the effectiveness of the environment. The Industrial Internet of Things (IIoT) indicates the use of classical concepts of the Internet of Things in industrial environments. By enabling the use of sustainable and efficient technologies in an industrial environment, IIoT enhances the manufacturing process.

At present, the IIoT market is experiencing a rapidly growing as well as increasingly accommodating market as part of the digital transformations of many industries. Large companies from around the world are investing in this emerging market due to the robust alliances and alignment of interests between IIoT stakeholders. The emerging market has attracted large companies from around the world because of the emerging applications [2]. A critical challenge for IIoT systems is security, because of the growing number of different departments and clients on IIoT systems. There are inherent vulnerabilities in IIoT systems arising from safety issues at different layers of IIoT [3]. The surveillance and analysis of the traffic can assist in managing networks and identifying security issues. Many national governments are investing in information and communications technology infrastructure to solve traditional public management problems. The implementation of a smart city is one of the most progressive and effective solutions. Transitioning from traditional public services and resources to a smart city model has several benefits, including improved service quality and reduced administrative expenses.

However, a strong network is required to manage government infrastructure in a smart city. Among the major issues facing smart environments in the real world are IoT security and complexity, as well as interoperability with other IoT technologies [4]. Therefore, several mechanisms have been suggested to find security issues in numerous domains. The intrusion detection system (IDS), as an effective information safety control system, has recently undergone an important development. Confidentiality, integrity, and availability are three crucial security concepts for programs and services in IIoT environments [5]. The IDSs have been widely used to identify harmful network traffic to defeat malicious behavior, especially when preventive approaches at the IoT endpoint fail. With the increasing complexity and furtiveness of hackers on IoT networks, improved intrusion detection methods are required to keep up with the changing threats [6,7,8].

Some kinds of unstructured data, including text, image, voice, video, etc. [9], cannot be processed using traditional machine learning (ML) methods [10]. IoT systems generate unstructured data that need to be processed by a powerful pattern recognition engine for anomaly detection in order to find and classify anomalies. Deep learning (DL) algorithms can be trained on a variety of data types [11]. In order to ensure that data are transferred securely and reliably in IoT networks, deep learning algorithms may be successfully deployed to discover anomalous behaviors in different IoT networks [12,13]. Adversarial machine learning (AML) applications on anomalies and malware are focused on the performance evaluation of detecting the new attacks [14]. The Generative Adversarial Network (GAN) has been frequently used in anomaly detection to combat adversarial perturbations from hackers [15]. Cybercriminals, for example, try to create typical data in order to trick the IDS into classifying the data in a bogus category [16]. However, one of the benefits of a GAN is that it can generate more training data in order to deal with unbalanced and missing data sets [17]. The GAN was able to amplify and enhance known and unknown adversarial disruptions in the context of anomaly detection, as well as strengthening the IDS against attacks. It also has a huge capacity for learning adversarial attacks in real-time streaming data, which aids the IDS in detecting malicious behavior [18].

As a result, the use of a GAN-based IDS aims to improve the feasibility and efficiency of the IDS in classifying normal and abnormal data, as well as reducing malicious supply chain risk management in the IoT security field, with the goal of detecting malicious activities on the network communication from unauthorized resources [19,20]. In addition, reinforcement learning (RL) is considered one of the most used methods recently in machine learning (ML) for solving complex high-dimensional datasets of intrusion detection. More precisely, the agent tries to learn in the environment to make decisions; then, when it estimates the reward, it can move to the next state. For example, the probability of each event can be modeled with the Markov decision process (MDP), which is based on the current event, to decide the next one. In a great deal of research, distributional RL performs the important task of finding the uncertainty of decisions taken by the MDP [21]. Unlike the traditional RL methods, where a value expectation function is trained, the distributional RL mechanisms preserve a full distribution of expected future returns [22].

In this paper, we combine the two approaches—the distributional RL and GAN—to overcome their disadvantages and propose a new classical method that combines the advantages of the two approaches and has enhanced performance over traditional methods. In order to improve the capability of learning and the efficiency of the IDS, we develop an improved framework for detecting anomalies in imbalanced data in the IIoT using the GAN. Data created by the GAN look and feel like real data. In contrast to the existing works in the literature, where the GAN agent is used either to create artificial attacks or for data augmentation, we show how the GAN agent is efficiently used to assist the distributional RL-based IDS to improve the detection of some minority attack types. To the best of our knowledge, we are the first to propose an IDS system based on DRL and GAN agents to work on the DS2OS dataset. Our main contributions are summarized as follows:

- We design and develop the RL and distributional RL as an intrinsic randomness process to find all possible returns from the immediate rewards and stochastic dynamics policy.
- We provide the GAN model for data balancing and data augmentation for the minor profiles.
- We build the proposed algorithm, which contains the returns of distributional RL using the synthetic data generated by the GAN to empower anomaly detection.
- We perform extensive experiments with the DS2OS dataset to validate the effectiveness of the DRL-GAN in binary and multi-class classification scenarios.
- We discuss the simulation results, which show that the proposed algorithm can improve the performance evaluation rate.

The rest of the paper is organized as follows: Section 2 provides related works that have focused on adversarial attacks in IoT environments. Section 3, describes the hybrid framework (DRL-GAN) proposed for anomaly detection in IIoT and discusses the data-collecting process for evaluating the model. Section 4 discusses the implementation results and the performance evaluation of the proposed scheme with the existing approaches. Finally, Section 5 articulates the conclusions of the paper and presents some future scopes.

## 2. Related Work

In this section, we describe literature research on the GAN based on adversarial ML/DL attacks on intrusion detection frameworks. We show the drawbacks associated with these contributions, which have motivated the suggested approach to avoid these issues. Hu et al. [23] proposed an approach to generate adversarial malware examples that apply a GAN-based algorithm called MalGAN for black-box attacks. They succeeded in generating adversarial malware samples to evade deep learning-based malware detection. Lin et al. [24] designed an IDSGAN framework for adversarial attack generation against the IDS. In their proposed scheme, a Wasserstein GAN is used to improve the generator and the discriminator, where the generator generates adversarial abnormal activities focusing on the evasion black-box and attacks the IDS. The authors generated the adversarial attacks in the KDDTest+ dataset, and the simulation results show the robustness of the IDSGAN. In the context of a wireless, self-organized ad-hoc network of cyber-physical systems, Belenko et al. [25] defined a machine-to-machine communication network used to connect cyber devices. These are cyber-physical devices that can be programmed to perform operations either by getting commands or remotely. In this way, an adequate and effective intrusion detection system is required to prevent the misuse of these devices by strangers. In order to cover this issue, an intrusion detection system is proposed in this study based on a GAN for large-scale cyber-physical systems (CPS).

Ferdowsi et al. [26] suggested a distributed adversarial network to provide a wholly decentralized IDS for the IoT area in order to detect anomalies, which is convenient for hiding the user’s sensitive data. Clements et al. [27] evaluated the vulnerability of the deep learning-based network intrusion detection system (DL-NIDS) to well-designed attacks from the domain of adversarial machine learning. This vulnerability is present in deep learning-based systems even when the model achieves a high degree of accuracy for classifying between benign and malicious network traffics. Therefore, researchers must take steps to verify the security of deep learning models in security-critical applications to ensure they do not impose additional risks. Yin et al. [28] proposed a GAN-based framework with the botnet detection model that enhances the performance of the detection mechanism for the most severe attacks while maintaining the key features of the original detection model. Ibitoye et al. [29] proved the impact of adversarial samples on deep learning based on IDS in the IoT network using feed-forward neural networks (FNN) and compared the results from several adversarial attacks with a self-normalizing neural network (SNN). The author showed that the DL-based IDS classifier utilizing FNN was negatively affected by the adversarial samples.

Shahriar et al. [30] developed a new security framework based on an IDS model using artificial neural networks and trained on data generated by a GAN. The use of the GAN in this work aims to overcome the issue of imbalance and missing sample data on emerging CPS technologies; the use of a standalone IDS is modeled and compared to the proposed GAN-IDS (G-IDS) through the NSL-KDD99 dataset, which showed that the proposed IDS outperforms existing trained IDS in the literature. Usama et al. [31] proposed an adversarial ML/DL attack using a GAN that can evade a black-box-based IDS and proposed defense procedures while ensuring the preservation of the function by modifying only the non-functional behavior of the adversary network traffic characteristics. The obtained results confirmed that the proposed scheme can be used to strengthen the IDS and make it more powerful against adversary disturbances. Pacheco and Sun [32] evaluated the efficiency of adversarial ML/DL attacks on the UNSW-NB15 and Bot-IoT datasets. They demonstrated the performance evaluation of adversarial attack algorithms including the Jacobian-based saliency map attack (JSMA), fast gradient sign (FGSM) method, and Carlini Wagner (CW) attack against ML classifiers such as the support vector machine (SVM), decision tree (DT), and random forest (RF). The experimental results indicate that the attacks were capable of successfully impairing the overall performance of the respective SVM, DT, and RF classifiers used on datasets.

Ullah and Mahmoud [33] proposed a framework for anomaly detection in IoT networks utilizing conditional GANs (cGANs) for the data unbalance and the binary class (bcGAN) for data enhancement. The performance evaluation of the model was tested utilizing a feed-forward neural network (FFN) on network-based anomaly detection datasets. Lee et al. [34] proposed a comparative study of the GAN-based anomaly detection (AD) methods including the MAD- GAN, the TAnoGAN, and the CUSUM chart. Zhao et al. [35] suggested an enhanced adversarial attack model based on the Wasserstein GAN named attackGAN. By adding the feedback from the IDS, the model can effectively perform an evasion attack and at the same time ensures the functionality of network traffic. The full set of their experiments was performed on the NSL-KDD dataset. Zhang et al. [36] designed a TIKI-TAKA framework to defend against adversary attacks on a DL-based Network IDS (NIDS) using multilayer perceptron (MLP), convolutional neural network (CNN), and CNN with long short term memory (LSTM) layers, named the C-LSTM-based network IDS, defending against adversarial malicious behaviors. Furthermore, they suggested incorporating the defense mechanisms of voting assembling, assembling adversarial training, and query detection to increase resistance to attacks. Jiang et al. [37] developed a feature grouping and multi-model fusion detector (FGMD) framework capable of defending against adversarial attacks by applying feature pooling and multi-model merging. Weinger et al. [38] discussed the problem of performance degradation in the federated learning (FL) setting in the context of class imbalance and device heterogeneity. The authors examined how data augmentation can be enforced to improve detection performance for IoT anomaly detection by conducting a thorough evaluation using different IoT datasets: TON-IoT and DS2OS datasets.

The contribution of our paper can be compared to existing approaches that demonstrate the use of a GAN for IoT security, as described in Table 1. However, none of these approaches addresses the issue of imbalanced and limited data. Unlike the works stated before, we suggest a thorough framework to combine training data that might enhance the effectiveness of IDS in detecting cyber attacks using a GAN. Although we tested our system using a Distributional Reinforcement Learning-based IDS and a recent intrusion detection dataset, it may be used with any IDS and a variety of industrial and network datasets.

## 3. Proposed Approach

### 3.1. System Model and Problem Formulation

In this paper, we describe our suggested model in detail . The main idea of the anomaly detection process is to resolve the problem of imbalanced data and improve the identification of each type of attack by applying machine learning methods.

**Figure 1:**
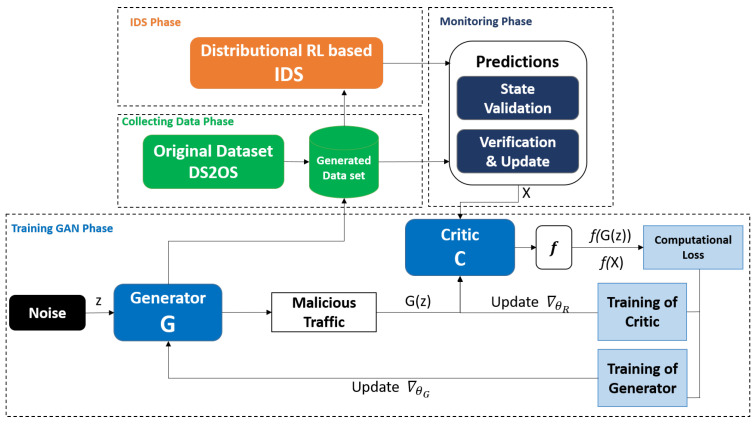
Process flow of anomaly detection based on DRL-GAN.

Figure 1 represents the anomaly detection’s architecture. It consists of two learning steps, which are outlined below. In this study, we offered to construct a robust IDS using a DRL-GAN approach. Our proposed DRL-GAN is an enhanced model with improved accuracy and minimal false alarms while using datasets with full features and increasing computational complexity.

### 3.2. Data Preparation Module

The selection of an appropriate dataset for assessing the anomaly detection system is crucial, which is why the data was selected before the simulation of the proposed approach was performed.

#### 3.2.1. Overview of Dataset

We have used the Distributed Smart Space Orchestration System (DS2OS) benchmark dataset gathered from Kaggle [39] as an open-source dataset provided by Pahl and Aubet [40]. A synthetic data set was collected from an IoT environment created virtually using DS2OS. This data set contains traces captured from various IoT simulation sites using different types of services, including light controllers, thermometers, movement sensor values, washing machines, battery and temperature status, and the manipulation of smart doors and smartphones. The dataset typically contains 357,952 data points with 347,935 and 10,017 normal and abnormal data points, respectively. The DS2OS dataset contains 13 features, described in Table 2, which can be classified into eight classes. These classes include normal data and seven types of attacks classified as Denial of Service, scan, malicious control, malicious operation, spying, data probing, and incorrect setup attacks. All of these classes in this data set are briefly described below:

- **Normal:** Normal data that are completely correct and accurate.
- **Denial of Service:** An attacker sends too many packets, flooding the target, and making the service unavailable to the server or other device.
- **Scan:** The system may be scanned to collect data through hardware, which can lead to data corruption.
- **Malicious control:** A software vulnerability could allow an attacker to obtain a valid session key or manage to capture network traffic. In this way, a malicious person can take control of the entire system.
- **Malicious operation:** These attacks are generally caused by malicious software. Malware refers to decoy activities that interfere with the original operation. This malicious operation can adversely affect the performance of the device.
- **Spaying:** An attacker exploits a vulnerability in the system to break into the system using a backdoor channel and discover important information. In any case, manipulating the data can be a major obstacle to the entire system.
- **Data probing:** In these types of attacks, malicious nodes create a different type of data instead of the original data.
- **Incorrect setup:** The incorrect system settings can cause data disruption.

The record distribution of training and testing sets in the DS2OS dataset is presented in Table 3.

#### 3.2.2. Data Preprocessing

To make computation easier, the extensive network traffic data from the DS2OS dataset were pre-processed to transform the features into appropriate formats. In this investigation, the data preprocessing included the following.

(A) **Collecting the data input** is the first important step in building the model’s feature selection. This process aims to identify a subset of suitable features that will lead the learning models to higher accuracy and robust detection. In the DS2OS analysis, we discovered some missing values of the type of continuous numerical “Accessed Node Type” included 148 values of “NaN” corresponding to abnormal values, and the categorical nominal value “Value” contained some data that were unaffected, such as “False”, “True”, “Twenty”, and “none” transformed into “0.0”, “1.0”, “20.0”, and “0.0”, respectively. Likewise, the feature “Timestamp” with the continuous numerical value was not considered in this study. Furthermore, the feature “Timestamp” with an ongoing numerical value was not considered in this study as it was removed from the train and test set of the DS2OS dataset to retain only 12 features.
(B) **Data encoding** refers to the process of transforming categorical “Nominal” data into vectors in such a way as to simplify the treatment task of the inputs and outputs of deep learning approaches. Since there are several paths to encode categorical values to learn the model, the most recommended schemes are label encoding, One Hot encoding, bin-counting, feature hashing, dummy coding, and effect coding techniques [41]. The DS2OS dataset includes nominal and categorical data. However, label encoding has been recommended to perform the conversion as it has the advantage of unifying the number of features; as a result, the dimension of the dataset does not increase.
(C) **Data normalization** has the benefit of making some machine learning algorithms faster. This phase is only recommended if the features have different value ranges. The purpose of this step is to change the values of the numerical columns of the dataset to a common scale without warping the differences in the value ranges.

### 3.3. Distributional Reinforcement Learning

The core of our proposed model is the distributional reinforcement learning-based IDS engine in which we rely on RL modeling from our previous work [42], and we briefly established the concept of *reinforcement learning* (RL) based on a *Markov decision process* (MDP). We briefly define the quintuple concepts of RL-based IDS by (*S*, *A*, *R*, $P_{a}$, γ) as follows:

- *S* represents the set of states captured by the IDS; we assume $S=\left\{s_{0},s_{1},s_{2}\right\}$, where $s_{0}$ denotes “*normal*”, $s_{1}$ “*Detection*”, and $s_{2}$ “*No Detection*”.
- *A* indicates the set of possible actions that can be taken by the IDS, which can be specified as *low*, *medium*, *high*, and *critical* as a reaction of the IDS according to the degree of risk of an attack [43].
- *R* is the objective function to be optimized in the system, which allows us to represent the returns of the IDS and to perform an action immediately with the location of reward R(s,a) received in the state *s* and the action *a*.
  (1) $$R\left(s,a\right)=\sum_{s^{\prime }\in S}P_{a}\left(s|s^{\prime },a\right)R\left(s\prime ,a\right)$$
- $P_{a}$ is the transition of state probability, modeled as a matrix of transition probabilities $p(s_{i}|s_{j},a)$ observed at time *t* for a∈A where i,j=1,2,3 and $V=\left\{1(valid),2(invalid)\right\}$
  (2) $$P_{a}\left(s_{t+1}=s_{j}|s_{t}=s_{i},a\right)=\sum_{j=1}^{3}\alpha_{i,j}\beta_{i,j}^{\left(a\right)},\;i=1,2,3$$
  where i,j∈V, $\beta_{i,j}^{a}$ represents the transition probability from the state $s_{j}$ observed at *t* to the state $s_{i}$ observed at t+1 in a 3 × 3 matrix $B_{a}$ with $\sum_{j=1}^{3}\beta_{i,j}^{a}=1$, wherein 0<α<1 with $\alpha \in \left[\frac{1}{2},1\right]$, $\alpha \in \left[0,\frac{1}{2}\right]$, respectively, describing the valid or invalid decision of predicted data.
- γ is the discount factor in 0<γ<1.

In each *s*, the agent realizes an action *a*, observes the reward *r* of this action as well as the next state as $s^{\prime }$ , and updates the estimated value function of $Q^{\pi }$ satisfying the Bellman equation [44]:

(3) $$Q^{\pi }\left(s,a\right)=E_{s,a,s^{\prime }}\left[R\left(s,a\right)+\gamma \max_{a^{\prime }\in A}(Q\left(s^{\prime },a^{\prime }\right)\right)]$$

This is done to give more predictions of actions by modeling all possible returns in a dynamic way while trying to learn from their mean.

Let the random variable $Z\left(s,a\right)=\sum_{i=0}^{\infty }\gamma R(s,a)$ be the return obtained by calculating the sum of discounted rewards observed by the agent by starting from state *s*, performing action *a* following the policy π. Thus, for a given π, the estimated value function is

(4) $$Q^{\pi }\left(s,a\right)=E\left[Z^{\pi }\left(s,a\right)\right]$$

The formulation of the distributional Bellman equation for a given π is represented as

(5) $$Z\left(s,a\right)\overset{D}{=}E_{s,a,s^{\prime }}\left[\left(R\left(s,a\right)+\gamma \max_{a^{\prime }\in A}Z^{\pi }\left(s^{\prime },a^{\prime }\right)\right)\right]$$

where the equation iterates $Z\leftarrow \tau^{*}Z$ and converges to $Z^{\pi }$, and $\overset{D}{=}$ means the distributional equivalence. However, to define the distributional Bellman optimality operator based on (5), for a given optimal policy $\pi^{*}$, we have

(6) $$\tau^{*}Z\left(s,a\right)\overset{D}{=}E_{s,a,s^{\prime }}\left[\left(R\left(s,a\right)+\gamma Z(s^{\prime },\pi^{*}\left(s^{\prime }\right))\right)\right]$$

where $s^{\prime }∼p(.|s,a)$ and $\pi^{*}={arg max}_{a^{\prime }\in A}E[Z(s^{\prime },a^{\prime })]$

### 3.4. Generator Adversarial Networks(GAN)

The GAN is a deep neural network model composed of two classes of machine learning models: generator *G* and discriminator *D* networks [45]. However, the generator *G* learns to generate falsified data as a random noise signal from a probability distribution p(z), which follows a normal distribution z∼N(0,1), and tricks the discriminator into accepting them as original input data. Afterwards, the discriminator *D* learns to differentiate between cheating data generated from *G* and the attacks found from the original data *X*. In addition, to train the stability of both *G* and *D*, we used gradient descent. By endorsing the formulation of the Wasserstein GAN (WGAN) and the notation of gradient penalty (GP) [46], the learning process of the WGAN is modeled by a min–max game between two players *G* and *C*, formulated as

(7) $$L=E_{\hat{x}∼P_{g}}\left[D\left(\hat{x}\right)\right]-E_{x∼P_{r}}\left[D\left(x\right)\right]+E_{\hat{x}∼P_{g}}\left[\lambda (|\right|\nabla_{\hat{x}}D\left(\hat{x}\right){\left|\right|}_{2}-1{)}^{2}],$$

Let us denote $p\left(\lambda \right)=\lambda (||\nabla_{\hat{x}}D\left(\hat{x}\right){\left|\right|}_{2}{-1)}^{2}$ as the gradient penalty and $\hat{x}=qx+\left(1-q\right)\hat{x}$, q∼U(0,1). The algorithm replaces the state-action value Q(s,a) by its distribution and uses WGAN-GP to learn the distribution over returns of the target of traffic data *X*. The generator *G* receives the current sensed data state *s*, and in each updated round, we sample a minibatch ${\left(s,a,r,s^{\prime }\right)}_{i=1}^{m}$ from *X* and use it to update the networks. The IDS implements the Bellman optimality operator *T* according to (7) and gets the samples of the real distribution, which can be calculated as

(8) $$X\left(t\right)=r\left(t\right)+\gamma^{b}\min G(z(t),s(t+1))$$

where the target generator network is *G*.

The loss functions of the generator *C* and critic *G* networks, respectively, are expressed as

(9) $$L_{C}=E_{\hat{x}∼P_{g}}[f\left(G^{a(t)}\left(z\left(t\right),s\left(t\right),\tau \left(t\right)\right)\right]-E_{x∼P_{r}}\left[f\left(X\left(t\right),\tau \left(t\right)\right)\right]+p\left(\lambda \right)$$

(10) $$L_{G}=E_{\hat{x}∼P_{g}}[f(G^{a(t)}\left(z\left(t\right),s\left(t\right),\tau \left(t\right)\right)]$$

### 3.5. Monitoring and Validation Aspect

This phase consists of deciding whether and how to use the GAN in the training process of the DRL-IDS system. The aim is to enhance the performance of the DRL agent in detecting attacks in terms of accuracy, precision, and F1 score. The impact of the GAN agent depends on the input data used to train the agent. For this, it is vital to define the type of data to feed the discriminator during the training. Thus, in the following, we consider three scenarios in which the GAN is trained on different classes of the DS2OS dataset. The obtained results are analyzed using the confusion matrices and classification report to determine the impact of the GAN. We must mention that the GAN is used based on the results of a simple DRL-IDS trained on the training set of the DS2OS dataset. Afterwards, the GAN which is already trained to generate a specific data class, is introduced in the training of the DRL agent to enhance the detection of this type of attack. The whole process of our proposed scheme is summarized in Algorithm 1.
**Algorithm 1:** The DRL-GAN algorithm
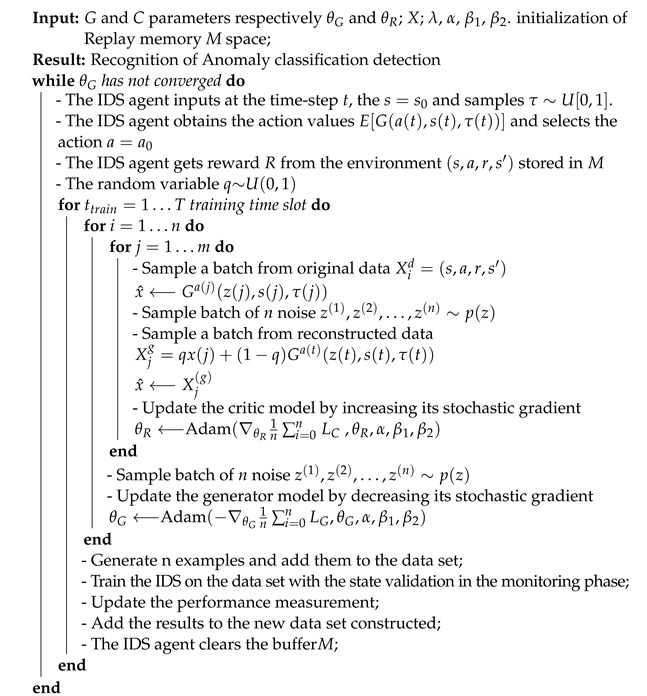


## 4. Results and Discussion

In this section, we present and analyze the results of the simulation performed to validate the effectiveness of the DRL-GAN model for anomaly detection in the Industrial IoT. The performance of the DRL-GAN model is compared with the normal DRL in binary and multi-class classification scenarios. Multiple GAN agents are considered in this study, where each of them is trained in a different class of the dataset. This is done to identify which scenarios the GAN can ameliorate the performance of the DRL-GAN.

### 4.1. Performance Metrics

Many performance metrics are accounted for when assessing the effectiveness of the offered method.

- **Accuracy:** The accuracy represents the percentage of normal and abnormal data that the IDS correctly predicted. Accuracy is expressed by
  (11) $$Accuracy\left(AC\right)=\frac{Tp+Tn}{Tp+Tn+Fp+Fn}$$
- **Precision:** The precision describes the ratio of normal recordings that are correctly detected by the IDS to all recordings that the IDS has recognized as normal. Precision is defined by
  (12) $$Precision\left(PR\right)=\frac{Tp}{Tp+Fp}$$
- **Recall:** The recall is the percentage of positive recording predicted correctly by the IDS. The recall is calculated as
  (13) $$Recall\left(RC\right)=\frac{Tp}{Tp+Fn}$$
- **F1-score:** The F1-score is calculated as the harmonic mean of the precision and recall metrics. The F1-score is determined by
  (14) $$F1\text{-}score\left(FM\right)=\frac{2Tp}{2Tp+Fp+Fn}$$

### 4.2. Performance Evaluation

We evaluate the performance of our proposed methods using the GAN agent in the training process to enhance the DRL-based IDS for detecting attacks. We suggested training the DRL agent on different data classes to analyze its impact. Firstly, we designed a DRL agent to detect attacks in IoT networks while the GAN agent is composed of the generator and discriminator. This latter is used only to train the generator and is introduced in the learning process of the DRL agent.

**Figure 2:**
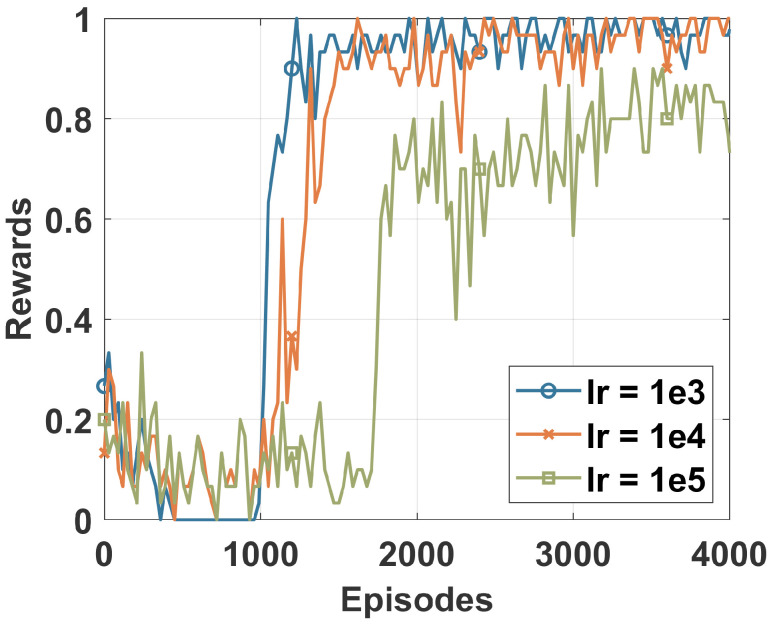
Training rewards of the DRL agent.

Figure 2 plots the cumulative reward of the DRL agent at each episode of the training process. The DRL agent was fed with a dataset sample at each episode to learn to identify different classes of the DS2OS dataset. Different learning rates were used to identify the most adequate for our study that provided stable learning for the agent. We can say that the learning rate of 0.0001 achieved the best convergence, as shown by the blue curve in Figure 2. The convergence of the DRL agent is achieved when its learning curve becomes flat and stops increasing. Only the results of the DRL trained using an LR of 0.0001 are used in the following.

**Figure 3:**
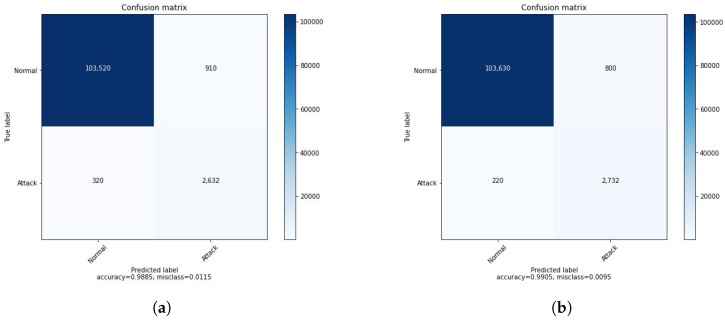
ConfusionMatrices of binary classification. (**a**) Normal DRL. (**b**) DRL with GAN.

Table 4 shows the results of the binary classification of the DRL agent before and after introducing the GAN agent in the training process. The GAN agent takes as input the attack samples and generates data to be used in the training of the DRL agent. The improved performance of the DRL agent is demonstrated by the second column of the accuracy. The impact of the GAN could be more clear and more significant when considering a multi-class classification. Matrices of confusion in Figure 3 support the results of Table 4 and show clearly how the GAN can enhance the IDS system. Before introducing the GAN, 320 attacks were classified as normal, presenting a huge amount of attacks not detected by the system. We can cope with this either by improving the DRL agent or enhancing the training process. We suggested introducing the GAN to generate additional data that help to improve the detection of some attacks. As shown in Figure 3, after applying the GAN, the number of not detected attacks was reduced by 100, demonstrating the importance of using the DRL.

**Figure 4:**
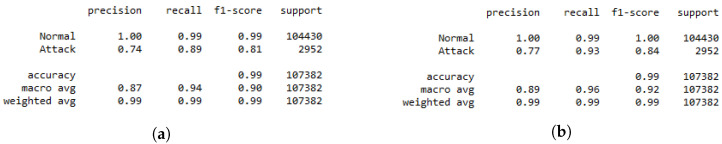
Binary classification report. (**a**) Normal DRL. (**b**) DRL with GAN.

The classification report presented in Figure 4 demonstrates how the DRL-based IDS system trained only using DS2OS can fail in identifying the attacks since its precision is about 0.74. This is due to unbalanced data resulting in an unstable learning process for the agent. After using the GAN agent in the training, the recall of the IDS is increased from 0.89 to 0.93, which is a significant improvement that ameliorates the performance of the system. However, the precision is improved by 0.03, which means that using the GAN in the training process of the binary classification system can be investigated further for better results.

**Figure 5:**
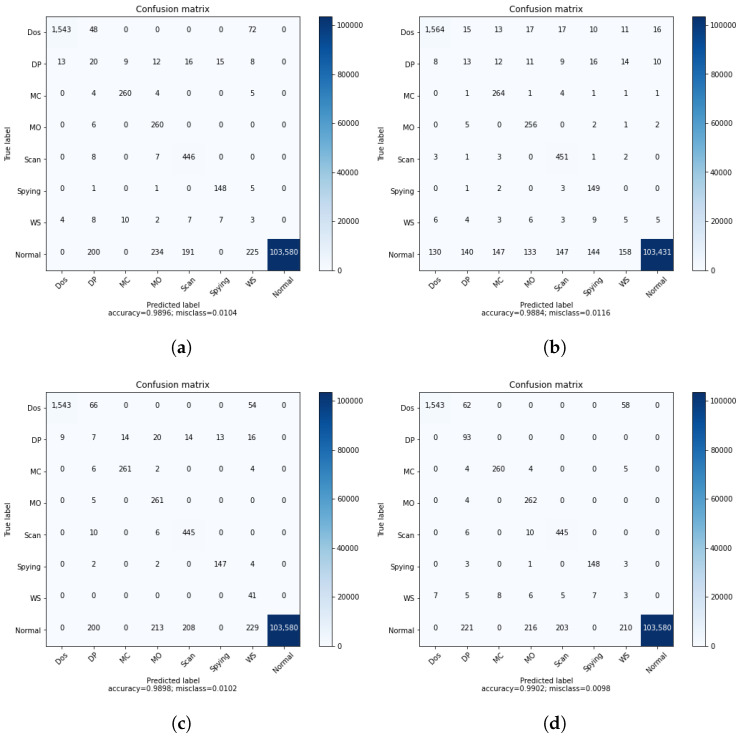
Confusion matrices of multi-class classification. (**a**) Normal DRL. (**b**) DRL with $GAN_{1}$. (**c**) DRL with $GAN_{2}$. (**d**) DRL with $GAN_{3}$.

**Figure 6:**
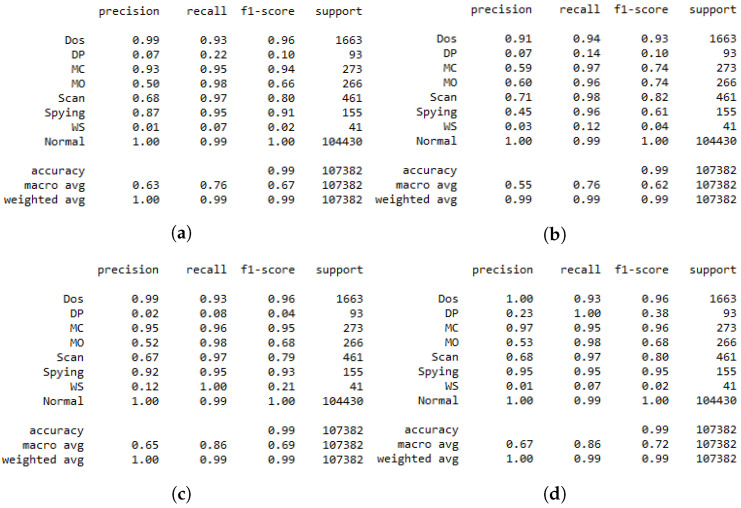
Multiclass classification report. (**a**) Normal DRL. (**b**) DRL with $GAN_{1}$. (**c**) DRL with $GAN_{2}$. (**d**) DRL with $GAN_{3}$.

**Figure 7:**
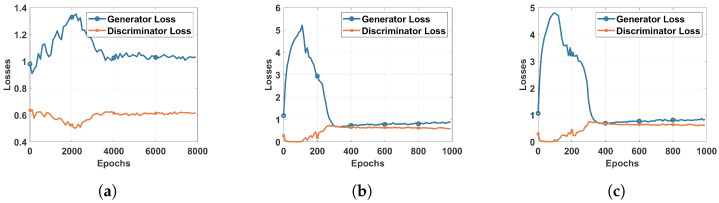
Losses of the Generator and Discriminator: (**a**) $GAN_{1}$ trained on full dataset (**b**) $GAN_{2}$ trained on WS class (**c**) $GAN_{3}$ trained on DP class.

However, the impact of the GAN agent can be shown clearly when considering the multi-class classification. For this, we trained a DRL agent on the DS2OS dataset using different learning rates to identify the most adequate rate, similarly to the process conducted for binary classification, as shown in Figure 2. The results of simple DRL without the GAN agent are shown by the confusion matrix and the classification report, respectively, in Figure 5a and Figure 6a. Those results helped us to train the proposed GAN agent and to identify its input. However, the results of the simple DRL show that data probing and incorrect setup attacks are not well detected by the trained agent, as we can see in the second and sixth rows. In addition, the classification report in Figure 6a shows that the precision of detecting data probing and incorrect setup attacks is about zero, which means that this system cannot recognize these attacks. This is due to a lack of samples presenting these attacks in the main dataset used for the training. Hence, the agent does not receive sufficient data to learn their feature combination and identify them in the inference process. We suggest introducing a GAN agent in the training process that generates artificial samples of the exact data type to enhance the dataset and ameliorate the learning process of the DRL agent. Three scenarios are considered in this study corresponding to the $GAN_{1}$, $GAN_{2}$, and $GAN_{3}$, where each one of these agents is trained on the DS2OS dataset part. $GAN_{1}$ is trained on the full dataset, $GAN_{2}$ is trained only on incorrect setup samples, and $GAN_{3}$ is trained on data probing samples. Figure 7 shows the loss convergence of the considered agents. Thus, the convergence of $GAN_{1}$ shown by Figure 7a seems to be the unequilibrated learning process, while both $GAN_{2}$ and $GAN_{3}$ trained on a specified dataset class show perfect convergence of their losses. This will impact the results of DRL using the GAN agent for the training process.

Table 5 shows the multiclass inference results of the DRL agent for different scenarios with and without a GAN agent in the training process. The aim of introducing the GAN is to improve the performance of the DRL-based IDS system, which can be seen in the second column of the accuracy. Where a simple DRL agent achieves an accuracy of 98.85, which is reduced when using the GAN trained in the full dataset, this means that $GAN_{1}$ perturbs the training process of the DRL agent since is not well trained, as discussed previously. Generating a full artificial dataset does not help the DRL agent and deteriorates the quality of the original dataset. On the other hand, $GAN_{2}$, trained on the WS class of the DS2OS dataset, enhances the accuracy by a simple, small amount. This is because of the small number of WS samples in the testing DS2OS dataset. In contrast, $DRL_{3}$, trained on the DP dataset, significantly improves the performance of the simple DRL in terms of the accuracy as well as the precision and the F1-score, respectively, in columns two, three, and four of Table 5.

Figure 5 represents the confusion matrices of multiclass classification using a DRL agent trained in different ways. As discussed above, Figure 5a shows the results of a simple DRL agent trained on the original DS2OS dataset without introducing the GAN agent. These results can be considered good for the proposed DRL-based IDS, although two attacks—namely, data probing and the incorrect setup—are not detected efficiently. This issue raises the need for using the GAN agent to enhance the dataset for training by generating an artificial dataset similar to the original. The GAN is trained to learn to generate artificial data similar to the input data of the discriminator. The efficient training of the GAN is shown by the loss convergence of both the generator and discriminator. The agent $GAN_{1}$ trained on the full dataset is not capable of improving the IDS system accuracy, since unstable artificial data were generated, which perturbed the learning of the agent and reduced its performance in testing, as shown in Table 5, second row. Afterwards, we focused on training the GAN agent on a specific class of the dataset targeting the generation of samples of the attacks missed by the DRL agent. In Figure 5c, we focused on improving the performance of DRL in detecting the incorrect setup by using the $DRL_{2}$ in the training process. This, as demonstrated in the confusion matrix in Figure 5c, helped the DRL agent to detect all attacks of type WS. In the same direction, we used the $DRL_{3}$ to improve the ability of the DRL agent to detect the DP attacks.

In Figure 6, we present the classification report of the DRL agent for multi-class classification where the performance of each class is provided separately. This helps to provide a clear insight into the impact of the GAN on the performance of DRL agents. The same scenarios as described above were considered in the same direction as the results in Figure 5, where the sample DRL agent results are presented by Figure 6. From this latter figure, we can easily identify that the two classes DP and WS are not identified by the DRL agent due to their low precisions, which are equal to 0.07 and 0.01, respectively. This is due to the unbalanced data due to the lack of some class data, resulting in an unstable learning process. Consequently, we suggested the use of the GAN agent in the training process of DRL to generate some artificial data, which help in performing stable learning. We can see from Figure 6b that the performance of the DRL deteriorated due to the use of the GAN trained on the full dataset. This is because a GAN fed by the whole data set including all the classes generates artificial data of the “Normal” class due to the important portion of this class in the training dataset. Thus, for the efficient use of the GAN agent, we proposed $DRL_{2}$ trained only on the WS class of data, aiming to help the DRL agent to detect this class in the test dataset, as shown in Figure 6c, where the recall of the WS attack improves from 0.07 to 1. Moreover, the agent $GAN_{3}$ trained on DP class data aims to improve the capability of DRL in detecting this class, as is shown by Figure 6d, where the recall of the DP class improves from 0.22 to 1, knowing that the recall presents the ratio of correct classification. By this, we can consider that a GAN trained on a single class of data can ameliorate the performance of DRL in detecting the specific data by generating artificial data of the target class to help in the training process. Table 6 shows the time spent for the training and inference of different proposed models. We can see from the table that the DRL incorporated with the GAN spends more time in the training process compared to the normal DRL, which is due to data augmentation introduced by the GAN. However, the same time duration was spent on the inference for the proposed model with and without the GAN agent.

## 5. Conclusions

This paper presents the design and development of Distributional Reinforcement Learning and Generative Adversarial Network-based anomaly detection models for Industrial IoT, namely DRL-GAN for an imbalanced dataset. The proposed model was evaluated using the DS2OS dataset in two model scenarios. Two models were used to assess the capability of DRL to detect all attacks, whereas the GAN model was used to generate the data augmentation. The normal DRL and DRL-GAN demonstrated their adaptability by achieving accurate identification. The performance of the normal DRL was compared to DRL-GAN, and it was found that the latter provides the best results on almost all evaluation criteria for multiclass and binary classifications. The consistent performance of the DRL-GAN model on the DS2OS data set proves the robustness of the proposed infrastructure for anomaly detection in the Industrial IoT. The proposed model composed of both distributional RL and a GAN agent suffers from the high computational resources required for the training process. In future work, we plan to further investigate anomaly detection in IoT networks through various optimizing techniques to enhance the learning ability of the DRL-GAN model and make it more efficient with smaller data samples.

## Figures and Tables

**Table 1:** Summary of the most relevant adversarial attacks based on IDS models in the literature.

Article	Year	Approach	Dataset	Performance Metrics	Main Contribution
Hu et al. [23]	2017	MalGAN	Standardized malware	TPR	A GAN-based algorithm (MalGAN) to generate adversarial malware examples to attack black-box malware.
Lin et al. [24]	2018	IDSGAN	NSL-KDD	DR, and Evasion Increase Rate (EIR)	Adversarial malicious traffic records generation against the IDS using Wasserstein GAN.
Belenko et al. [25]	2018	ANN	Ian Goodfellow	-	A generative adversarial ANN to detect anomalies in large-scale networks of cyber-physical systems (CPS).
Ferdowsi et al. [26]	2019	SBHAR	AC, PR, and FPR	AC, PR, and FPR	A distributed GAN-based IDS model to detect anomalous behaviors in IoT.
Clements et al. [27]	2019	DL-NIDS	Kitsun	FPR, and FNR	Vulnerability of DL-NIDS to well-designed attacks in the field of adversarial machine learning.
Yin et al. [28]	2019	Bot-GAN	ISCX botnet	AC, PR, FPR, and FM	A framework based on GAN to enhance botnet detection models (Bot-GAN).
Ibitoye et al. [29]	2019	FNN, SNN	Bot-IoT	AC, PR, FPR, FM, MC coefficient, and Cohen Coppa Score	Analyzing adversarial attacks against Feed-Forward Neural Networks (FNNs) and the Self-Normalizing Neural Network (SNN).
Shahriar et al. [30]	2020	G-IDS	NSL-KDD	PR, RC, and FM	A GAN-based intrusion detection system (G-IDS) for detection attacks in cyber-physical systems (CPS) technologies.
Usama et al. [31]	2020	GAN	KDD Cup 99	AC, PR, RC, and FM	An adversarial ML attack using generative adversarial networks (GANs) to evade the vulnerability of ML algorithms in network IDS.
Pacheco et Sun [32]	2021	MLP, SVM, RF, DT	UNSW-NB15 and Bot-IoT	AC, RC, FM, ROC, and AUC	Evaluation of the effectiveness of adversarial deep learning attacks against contemporary datasets.
Ullah et Mahmoud [33]	2021	cGAN	KDD’99, NSL-KDD, BoT-IoT	AC, PR, RC, TNR, FNR, FPR, and FM	A framework for detecting anomalies in IoT networks using conditional GANs (cGANs).
Lee et al. [34]	2021	MAD-GAN, TAnoGAN	SWaT data	AC, PR, RC, FPR, and FM	Anomaly detection for time series using MAD-GAN and the TAnoGAN.
Zhao et al. [35]	2021	attackGAN	NSL-KDD	DR	An improved adversarial attack model based on a Generated Adversarial Network.
Zhang et al. [36]	2022	Tiki-Taka	CSE-CIC-IDS2018	AC, PR, RC, and FM	A framework for defending against adversarial attacks on deep learning-based NIDS.
Jiang et al. [37]	2022	FGMD	IoTID, MedBIo	AC, PR, RC, FPR, and FM	An FGMD (Feature Grouping and Multi-Model Fusion Detector) framework against adversarial attacks.
Weinger et al. [38]	2022	FL	TON-IoT and DS2OS	AC, PR, RC, and FM	Improving detection performance for IoT anomaly detection (AD) using Federated Learning (FL).
Our contribution	2022	DRL-GAN	DS2OS	AC, PR, RC, FPR, and FM	Enhance the detection of anomalies and resolve the imbalance data problems in IIoT using DRL-GAN.

**Table 2:** DS2OS dataset features.

Feature	Type
Accessed node type	Nominal
Accessed node address	Nominal
Destination services address	Nominal
Destination services type	Nominal
Destination location	Nominal
Source ID	Nominal
Source address	Nominal
Source type	Nominal
Source location	Nominal
Normality	Nominal
Operation	Nominal
Value	Continuous
Timestamp	Discrete

**Table 3:** Class distribution of DS2OS dataset.

Attack Type	Training Set	Testing Set	Total
Normal	260,951	86,984	347,935
DoS	4335	1445	5780
Scan	1160	387	1547
Malicious control (MC)	667	222	889
Malicious operation (MO)	604	201	805
Spying	399	133	532
Data probing (DP)	257	86	342
Wrong setup (WS)	92	31	122

**Table 4:** Binaryclassification.

Proposed Schemes	Accuracy	Precision	F1 Scorel
**Normal DRL**	98.854557	98.994024	98.904968
**DRL with GAN**	99.050120	99.171315	99.091281

**Table 5:** Performance evaluation of multi-class classification for different scenarios.

Proposed Schemes	Accuracy	Precision	F1 Score
**Normal DRL**	98.955132	99.565517	99.222398
**DRL with $GAN_{1}$**	98.836863	99.312751	99.026353
**DRL with $GAN_{2}$**	98.978414	99.585785	99.234788
**DRL with $GAN_{3}$**	99.024045	99.620359	99.269372

**Table 6:** Computational time for training and prediction of different scenarios.

Approaches	Training Time (s)	Predicting Time (s)
**Normal DRL**	1101.45	0.52
**DRL with $GAN_{1}$**	1355.13	0.54
**DRL with $GAN_{2}$**	1367.70	0.53
**DRL with $GAN_{3}$**	1361.48	0.54

## Data Availability

The data presented in this study are available on request from the corresponding author.

## Notation

The following notation of parameters are used in this manuscript:

*G*	Generator network
*C*	Critic network
$\theta_{G},\theta_{R}$	Parameters of generator or discriminator
$\alpha ,\beta_{1},\beta_{2}$	Parameters of Adam optimizer
$S_{t}$	State captured by the agent at time-slot *t*
$A_{t}$	Possible actions taken by the agent at time-slot *t*
$R_{t}$	Reward returned to the agent at time-slot *t*
$P_{a}$	Transaction of state probability matrix
γ	Discount factor, where 0<γ<1
τ	Bellman operator
*X*	Original Data
*M*	Replay memory data set
*z*	Random noise vector
λ	Coefficient of penalty
